# Metabolomic and lipidomic plasma profiles according to metabolic dysfunction-associated steatotic liver diseases (MASLD) stages in the absence of type 2 diabetes (T2D).

**DOI:** 10.1007/s11306-026-02437-1

**Published:** 2026-05-07

**Authors:** Marion Pradeau, Julie-Catherine Coll, Ana Berteaux, Véronique Paquet, Isabelle Robillard Frayne, Stéphanie Ferland, Matthieu Ruiz, Anne-Marie Carreau

**Affiliations:** 1 Axe Endocrinologie-Néphrologie, Centre de recherche du CHU de Québec, Université Laval, Québec, QC Canada; 2 Département de Médecine, Faculté de Médecine, Université Laval, Québec, QC Canada; 3 Metabolomic Platform, Montreal Heart Institute Research Center, Montréal, QC Canada; 4 Département de Nutrition, Université de Montréal, Montréal, QC Canada

**Keywords:** Metabolomics, Lipidomics, Fibrosis, Metabolic dysfunction-associated steatotic liver disease

## Abstract

**Introduction:**

: Amino acids (AAs), tricarboxylic acid (TCA) cycle intermediates, and acylcarnitines (ACs) can reflect energetic metabolism. Metabolic dysfunction-associated steatotic liver (MASLD) has been associated with the modification of plasma AAs, ACs and TCA cycle intermediates’ profiles, but the changes in advanced fibrosis without type 2 diabetes (T2D) are not well studied.

**Objectives:**

The objective of this pilot study was to describe the targeted plasma metabolomic profile in individuals with advanced fibrosis to test research hypotheses concerning hepatic energy metabolism. Methods: We compared plasma fasting concentrations of 21 AAs, 11 organic acids (including ketone bodies and TCA cycle intermediates) and 14 ACs between individuals with advanced fibrosis stages (F3-F4/4) (*n* = 10) and individuals with no advanced fibrosis (*n* = 10), all without T2D and with similar clinical characteristics.

**Results:**

Median age (IQR) (51 [43–67] vs. 57 [43–66] years), sex (30 vs. 50% men) and BMI (35 [28–37] vs. 37 [32–39] kg/m^2^) were comparable between groups. The advanced fibrosis (AF) group presented higher plasma tyrosine (*p* = 0.04), α-ketoglutarate (*p* = 0.04), and a lower level of medium-chain ACs C8 and C10 (*p* = 0.04). The glutamate-glycine-serine (GSG) index, which combines AAs involved in glutathione metabolism, was higher in the AF group (*p* = 0.04).

**Conclusion:**

Overall, our results suggest impaired AAs catabolism and mitochondrial dysfunction. While the limited sample size and study design preclude causal inferences, these findings highlight potential metabolic signatures of advanced fibrosis in MASLD. They also underscore the need for larger, longitudinal studies to clarify their origin, significance, and clinical implications.

**Supplementary Information:**

The online version contains supplementary material available at 10.1007/s11306-026-02437-1.

## Introduction

Metabolic dysfunction-associated steatotic liver diseases (MASLD) are the most common liver diseases worldwide, affecting up to 38% of the population (Younossi et al., 2023). Among patients with excess liver fat (> 5% of hepatocytes), it is estimated that 3.3% of them are affected by the most severe form of the disease, with a mortality rate of 7.9% per year (Owrangi et al., 2025). However, the progression from non-advanced stages (with a fibrosis score between F0 and F2) to advanced forms (F3-F4) of MASLD is poorly understood. According to preclinical models, the progression of fibrosis could be driven by metabolic factors, including impaired hepatic anaplerosis/cataplerosis leading to increased tricarboxylic acid (TCA) cycle activity (Sunny et al., 2011), and mitochondrial dysfunction (Shum et al., 2021).

Mitochondrial function has been hypothesized to change during the onset and progression of MASLD, but its study in humans at advanced stages of MASLD is lacking (Fromenty & Roden, 2023; Shum et al., 2021). The general current hypothesis is that, in the first stage of the disease, hepatic mitochondrial oxidative capacity is increased, leading to the production of reactive oxygen species (ROS), subsequent oxidative stress and inflammation. At the advanced fibrotic stage, inflammation and oxidative stress continue to increase, but mitochondrial oxidative capacity decreases. These mitochondrial disturbances could lead to an increase in short and medium-chain acylcarnitines (ACs) (Li et al., 2019; Zhao et al., 2020). In addition, some amino acids (AAs) have an anti-oxidant role via glutathione synthesis (Chandel, 2021).

Studies suggest that tyrosine, glycine, glutamate and serine could help identify the presence, the severity and the stage of progression of MASLD (Gaggini et al., 2018; Gobeil et al., 2022). AAs also play a role in metabolic energetic pathways, such as the TCA cycle. Indeed, AAs can be converted into TCA cycle intermediates to replenish its reserves; this process is named anaplerosis (Owen et al., 2002). TCA cycle intermediates have been studied as non-invasive markers of mitochondrial dysfunction in the MASLD context (Sandlers et al., 2020). Plasma AAs and TCA intermediates concentrations were never assessed in advanced fibrosis, but may help to evaluate alterations in energy metabolism associated with advanced stages of MASLD.

One of the main gaps in metabolomics studies in MASLD is the presence of many confounders, mainly type 2 diabetes (T2D), insulin resistance, and obesity that are concomitant to MASLD. The objective of this pilot study was to assess targeted metabolomics that would support hypotheses on pathophysiological metabolic pathways involved in MASLD advanced liver fibrosis in the absence of T2D.

## Materials and methods

### Participants selection

Participants were consecutively voluntarily enrolled in a prospective observational study if they attended the multidisciplinary MASLD clinic at CHU de Québec-Université Laval, as previously described (Pradeau et al., 2025). All participants gave written informed consent. This study was approved by the ethics committee of the Centre de recherche du CHU de Québec – Université Laval (n°2021–5285) and was conducted in conformity with the Helsinki Declaration. Liver fibrosis evaluation was assessed as part of the usual clinical care of the patients, i.e., using non-invasive measurements, imaging, or biopsy, as described previously (Pradeau et al., 2025).

Among the 199 participants included in the prospective study, 99 completed a study visit with blood draw; and 20 participants without T2D, matched for age, sex, and BMI, were selected and separated equally into a group with advanced fibrosis (AF: F3-F4/4) and a group without advanced fibrosis (No-AF: F0-F2/4) (absence of evidence of advanced fibrosis).

### Sample collection and biological assays

The blood samples were collected after a minimal 8 h-fast, during the research visit. Blood was collected in ethylenediaminetetraacetic acid (EDTA) collection tubes and centrifuged a + 4 °C for 10 min at 3 800 rpm.

Biological assays, such as fasting glucose and insulin, glycated hemoglobin (HbA1c), liver enzyme and lipid panel were realized with the same methodology as previously described (Pradeau et al., 2025).

### Anthropometric measurements

Standardized procedures were used to record height, weight, and waist and hip circumferences. Body composition was assessed through bioelectrical impedance analysis (InBody^®^ 270, InBody Canada, Ottawa, Canada).

### Gas chromatography-mass spectrometry-based analysis of organic acids and amino acids

Procedures have been previously described (David et al., 2025; Lauzier et al., 2013; Vaillant et al., 2016). Briefly, one hundred µL of plasma samples were extracted with 70% methanol and 1 mol/L hydroxylamine (at pH 7.6). Isotope-labelled internal and external standards were added as follows: 400 nmol ^13^C_3_-lactic acid, 20 nmol ^13^C_3_-pyruvic acid, 20 nmol ^13^C_4_-β-hydroxybutyric acid, 1 nmol ^13^C_4_-α-ketobutyric acid, 20 nmol D_4_-citrate, 20 nmol ^13^C_4_-α-ketoglutaric acid, 20 nmol D_4_-succinic acid and 40 nmol D_3_-malic acid, 20 nmol ^13^C_4_-acetoacetic acid, 70 nmol ^13^C_3_-alanine, 50 nmol ^13^C_2_-glycine, 50 nmol ^13^C_5_-valine, 25 nmol ^13^C_6_,^15^N-leucine, 20 nmol ^13^C_6_-isoleucine, 40 nmol ^13^C_5_,^15^N-proline, 5 nmol ^13^C_5_-methionine, 20 nmol ^13^C_5_,^15^N-serine, 25nmol ^13^C_4_,^15^N-threonine, 10 nmol D_5_-phenylalanine, 2 nmol ^13^C_4_, ^15^N-aspartic acid, 20 nmol ^13^C_5_,^15^N-glutamic acid, 20 nmol ^13^C_6_-arginine, 10 nmol ^13^C_9_-tyrosine, 10 nmol ^13^C_6_-histidine, 80 nmol ^13^C_5_,^15^N_2_-glutamine, 15 nmol ^13^C_4_,^15^N_2_-asparagine, 10 nmol ^13^C_11_,^15^N_2_ tryptophan, 50 nmol ^13^C_3_,^15^N cysteine, 30 nmol ^13^C_6_,^15^N_2_ lysine. After 2 min in a sonication bath, 1 mol/L hydrochloric acid was added to the samples before an incubation at 70 °C for 15 min and then centrifuged at 22 000 g for 10 min. The supernatants were collected and evaporated to near dryness under a nitrogen flow. The samples were then rinsed once with 100% methanol and dried twice with ammonium sulfate. After centrifugation at 7 000 g, the supernatants were evaporated again to near dryness to transfer them into Gas chromatography-Mass spectrometry (GC-MS) vials. They were then evaporated to dryness. Finally, samples were resolubilized in pyridine at 45 °C for 90 min, followed by derivatization using *N*-methyl-*N*-tertbutyldimethylsilyltrifluoroacetamide at 90 °C for 4 h. Samples were injected into a gas chromatograph (Agilent 6890 N), with helium as carrier gas, coupled to a mass spectrometer (Agilent 5975 N) operating in electronic ionization (EI) mode at a flow rate maintained throughout (7.7 mL/min) in split mode (5.6:1). The temperature program was fixed as follows: 150 °C for 3 min, increment of 7 °C/min up to 210 °C and maintained constant for 3 min, increment of 7 °C/min up to 310 °C and maintained for 5.5 min and then 40 °C/min up to 320 °C. Metabolites were identified according to their m/z and retention time and quantified using internal or external standards as well as standard curves. Quality control data are available in Supplemental Table S1.

### Liquid chromatography-mass spectrometry-based analysis of acylcarnitines

A stock solution standard mix was prepared extemporaneous to give final quantities of 4.5 nmoles d9-C0-Car, 1.125 nmoles d3-C2-Car, 75 pmoles d3-C3-Car, 30 pmoles d3-C4-Car, 15 pmoles d3-C8-Car, 7.5 pmoles d3-C12-Car and 4.5 pmoles M + 4-C16-Car. As previously described (Ruiz et al., 2019), a modified protocol was developed from Peng *et al. (*Peng et al., 2013). Plasma samples were prepared as follow: 100 µl plasma and 46.7 µl of the standard mix were added to 2.5 ml acetonitrile, then vortexed for 2 min. The mixtures were frozen at -20 °C for 20 min and centrifuged at 7 500 g for 10 min. The supernatants were collected in 8 × 75 mm glass tubes. A second extraction was performed by addition of 500 µl ethyl ether. Mixtures were vortexed 2 min and centrifuged at 7 500 g for 10 min. The supernatants were collected in the same glass tube before evaporation under nitrogen gas until 0.5 ml, transferred in a screw vial with fixed insert (Alwsa Technologies) and evaporated until dryness. Seventy-five µl of methanol was added, samples were vortexed 50 s, 25 µl of water was added and samples were vortexed once again 50 s. The samples were centrifuged at 4 000 rpm for 5 min before transferred in new vials.

An Agilent Technologies ultra-high-performance liquid chromatography (UHLC) 1290 was used for separation of free carnitine and acylcarnitines. Four µl of sample kept at 8 °C were injected and separation was performed on an Atlantis dC18 silica column (50 mm x 4.6 mm, particle size 3µM, Waters) with a temperature maintained at 30 °C. Two different eluents were used for the mobile phase: eluent A (water/0.2% formic acid (FA)/10mM Ammonium Formate (AF)) and eluent B (95 acetone/ 5 Methyl tert-butyl ether (MTBE), 0.2% FA). Elution began with 100% eluent A for 4.5 min at a flow rate of 0.5 ml/min. Then eluent B is increased up to 50% until 33 min and up to 100% from 43 min to 69 min. At 70 min, the gradient was returned to 100% eluent A and 5 min was necessary to re-equilibrate the column. The flow rate was maintained at 0.5 ml/min during the 70 min total run time. A 6495 Triple Quad MS/MS system equipped with an ESI Agilent Jet Stream source (Agilent Technologies) was used. Nitrogen gas was used as collision gas and analytes were monitored in positive dynamic multiple reaction monitoring (MRM) mode (dMRM). The MRM parameters were optimized for standard molecules using the Mass Hunter Optimizer (version B05, Agilent Technologies). For acylcarnitines without pure standards, the fragmentor and the collision energy values were assigned following the observed pattern with standard molecules. The parameters fixed on the source were: gas temperature, 290 °C; gas flow, 11 l/min; nebulizer source gas, 35 psi, sheath gas temperature, 375 °C; sheath gas flow, 12 l/min; capillary voltage, 3 500 V and nozzle voltage, 0 V.

Peak integrations were performed with Mass Hunter QQQ quantitative (version B.07) and qualitative (version B.07) software. Semi-quantitative analysis was done by normalizing to the selected internal standards. Upon fragmentation, the m/z product ion 85 of all the different species served for quantification via ratio intensities and m/z qualifier 60 was used to validate identity and purity of the compounds.

### Statistical analyses

Results for continuous variables are expressed as median (25–75 percentiles). Normality of data was assessed with the Shapiro-Wilk test, and homogeneity of variance with the Levene test. As some data did not follow a normal distribution, nonparametric tests were performed on the entire dataset. Statistical differences were calculated with the Mann-Whitney U test or the Fisher’s exact test when appropriate. Analyses were performed on GraphPad Prism^®^ V.8.4.3 (GraphPad Software, La Jolla, CA) and R-Studio Version 2024.12.0 + 467 (The R Foundation for Statistical Computing, Vienna, Austria). P-values ≤ 0.05 were considered statistically significant. Because of the small sample size, we also reported all p-values ≤ 0.1. Because of multiple comparisons, Mann-Whitney p-values were adjusted using the Benjamini-Hochberg method and are reported in the text as FDR.

## Results

### Participants characteristics

A total of 20 participants were included in this analysis. Participants’ characteristics of each group are presented in Table 1. Age, BMI, percent body fat, lean body mass, waist circumference, and waist-to-hip ratio were comparable between the two groups. Detailed pharmacological treatments and comorbidities are also presented in Table 1. Fasting glucose and glycated hemoglobin (HbA1c) were higher in the advanced fibrosis (AF) group without reaching statistical or clinical significance. Fasting insulin was significantly higher in the AF group (*p* = 0.05). Both groups were insulin resistant, and insulin resistance indices were statistically comparable between groups (HOMA2-IR, HOMA2%S and ADIPO-IR), but exhibited light clinical differences. Liver enzymes were not different between groups except for the AST/ALT ratio, which was significantly higher in the AF group (*p* = 0.005). Lipid profiles were similar between groups (*p* ≥ 0.22). 95% of participants were of European descent.

**Table 1:** Participants’ characteristics (*n* = 20)

Parameters	No-advanced Fibrosis (No-AF) (F0, F1, F2) (*n* = 10)	Advanced fibrosis (AF) (F3-F4) (*n* = 10)	*p*-value
Age (years)	51.22 [42.89–66.84]	56.59 [43.10–66.30]	0.85
Sex (men in %)	3 (30)	5 (50)	0.65
Anthropometric measurements			
Body weight (kg)	95.5 [74.4-106.5]	105.1 [87.6-111.1]	0.32
Body mass index (kg/m^2^)	34.87 [28.11–36.91]	36.7 [31.93–38.89]	0.32
Percent body fat (%)	42.40 [35.43–51.10]	38.30 [33.10–48.40]	0.44
Lean body mass (kg)	55.25 [43.98–62.63]	55.40 [39.40–71.50]	0.58
Waist circumference (cm)	110.3 [100.9-117.6]	117.3 [109.7-124.5]	0.22
Hip circumference (cm)	117.0 [95.09–124.7]	119.0 [110.7-126.2]	0.58
Waist-to-hip ratio	0.93 [0.91–1.01]	1.01 [0.91–1.05]	0.35
Laboratory assays and indices			
Fasting glucose (mmol/L)	5.4 [5.18–5.58]	6.1 [5.3–6.85]	0.08
Fasting insulin (pmol/L)	173.2 [131.5-220.3]	235.6 [185.2-365.3]	**0.05**
HbA1c (%)	5.25 [5.08–5.4]	5.55 [5.18–5.8]	0.07
HOMA2-IR	3.18 [2.46–4.05]	4.22 [3.28–5.95]	0.12
HOMA2%S	31.5 [25.28–40.78]	23.75 [17.6-30.75]	0.12
HOMA2%B	102.4 [82.48–138.6]	92.20 [74.1–115.0]	0.39
ADIPO-IR	64.38 [38.57–88.99]	90.24 [67.46–156.0]	0.12
AST (U/L)	23.5 [18.0–42.0]	36.0 [23.5-53.75]	0.15
ALT (U/L)	32.0 [24.25–85.25]	41.5 [29.25–54.25]	> 0.99
AST/ALT ratio	0.65 [0.5–0.83]	0.97 [0.8–1.03]	**0.005**
Total cholesterol (mmol/L)	4.34 [3.78–5.18]	4.00 [3.54–4.52]	0.22
Triglycerides (mmol/L)	1.72 [1.31–2.35]	1.48 [0.97–2.27]	0.67
HDL (mmol/L)	1.25 [0.95–1.47]	1.06 [0.95–1.51]	0.80
LDL (mmol/L)	2.23 [1.91–3.15]	1.92 [1.41–2.81]	0.36
Comorbidities			
Arterial hypertension (%)	4 (40)	4 (40)	> 0.99
Hypertriglyceridemia (%)	0 (0)	1 (10)	> 0.99
Hypercholesterolemia (%)	1 (10)	3 (30)	0.58
Prediabetes (%)	3 (30)	3 (30)	> 0.99
Medications			
GLP1-RA (%)	1 (10)	1 (10)	> 0.99
Metformin (%)	2 (20)	3 (30)	> 0.99
Statin (%)	2 (20)	3 (30)	> 0.99
ACEI or ARA(%)	0 (0)	2 (20)	0.47
Calcium channel blockers (%)	3 (30)	3 (30)	> 0.99
Beta blockers (%)	1 (10)	1 (10)	> 0.99
Thiazides (%)	1 (10)	1 (10)	> 0.99
Diagnostic modality			
Clinical/biochemical (%)	4 (40)	0 (0)	
Fibroscan (%)	2 (20)	7 (70)	
Biopsy (%)	4 (40)	3 (30)	

Data are presented as median (25 and 75 percentiles) for continuous variables or as n (%) for discrete variables. A Mann-Whitney U test was performed for continuous variables, and a Fisher’s exact test was performed for discrete variables. *ACE* Angiotensin-converting enzyme inhibitors, *ADIPO-IR* Adipose tissue insulin resistance index, *AF* Advanced fibrosis, *ALT* Alanine aminotransferase, *ARA* angiotensin II receptor blockers, *AST* Aspartate aminotransferase, *GLP1-RA* Glucagon-like peptide-1 receptor agonists, *HbA1c* glycated hemoglobin, *HLD* High density lipoprotein, *HOMA2* Homeostasis Model assessment 2, *LDL* Low density lipoprotein, *No-AF* No advanced fibrosis

### Plasma amino acids

Twenty-one AAs were measured in the plasma of participants, and all data (Median [25–75]) are presented in Supplemental Table S2.

**Fig. 1:**
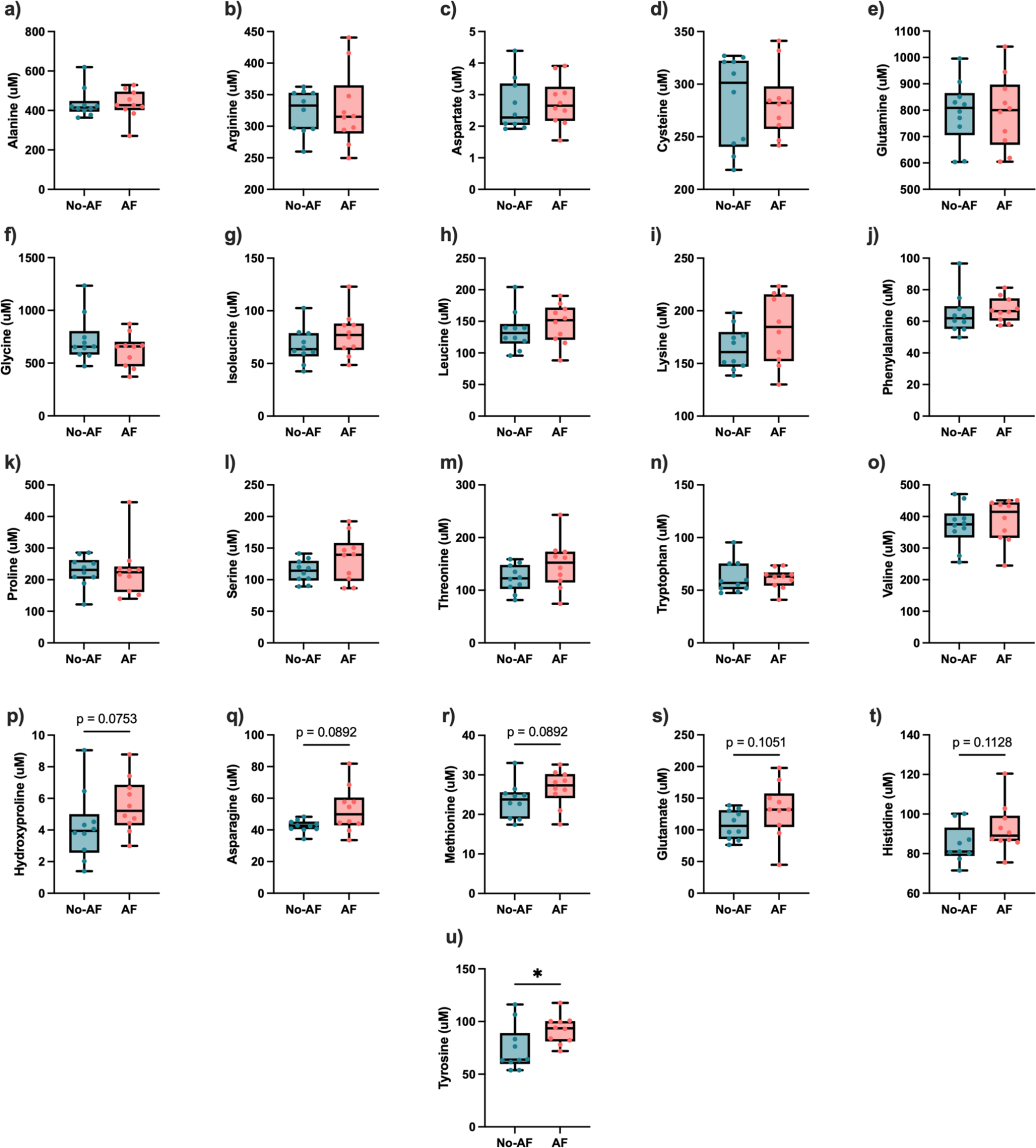
Increase of some amino acids, mainly tyrosine, in the AF group. Plasmatic concentration of **a** alanine, **b** arginine, **c** aspartate, **d** cysteine, **e** glutamine, **f** glycine, **g** isoleucine, **h** leucine, **i** lysine, **j** phenylalanine, **k** proline, **l** serine, **m** threonine, **n** tryptophane, **o** valine, **p** H-hydroxyproline, **q** asparagine, **r** methionine, **s** Glutamate, **t** histidine and, **u** tyrosine in No-Advanced Fibrosis (No-AF) and Advanced Fibrosis (AF) groups. Median with interquartile range, *p < 0.05, Mann-Whitney test

Individually, alanine, arginine, aspartate, cysteine, glutamine, glycine, isoleucine, leucine, lysine, phenylalanine, proline, serine, threonine, tryptophan and valine were not statistically different between groups (*p* ≥ 0.17, FDR ≥ 0.74) (Fig. 1a to 1o)**.** Hydroxyproline, asparagine, methionine, glutamate and histidine were higher in the AF group but did not reach statistical significance (*p* = 0.08, FDR = 0.62; *p* = 0.09, FDR = 0.62; *p* = 0.09, FDR = 0.62; *p* = 0.1, FDR = 0.65; *p* = 0.1, FDR = 0.62, respectively) (Fig. 1p to 1t). Only the plasma tyrosine concentration was statistically higher in the AF group (*p* < 0.05, FDR = 0.57) (Fig. 1u).

**Fig. 2:**
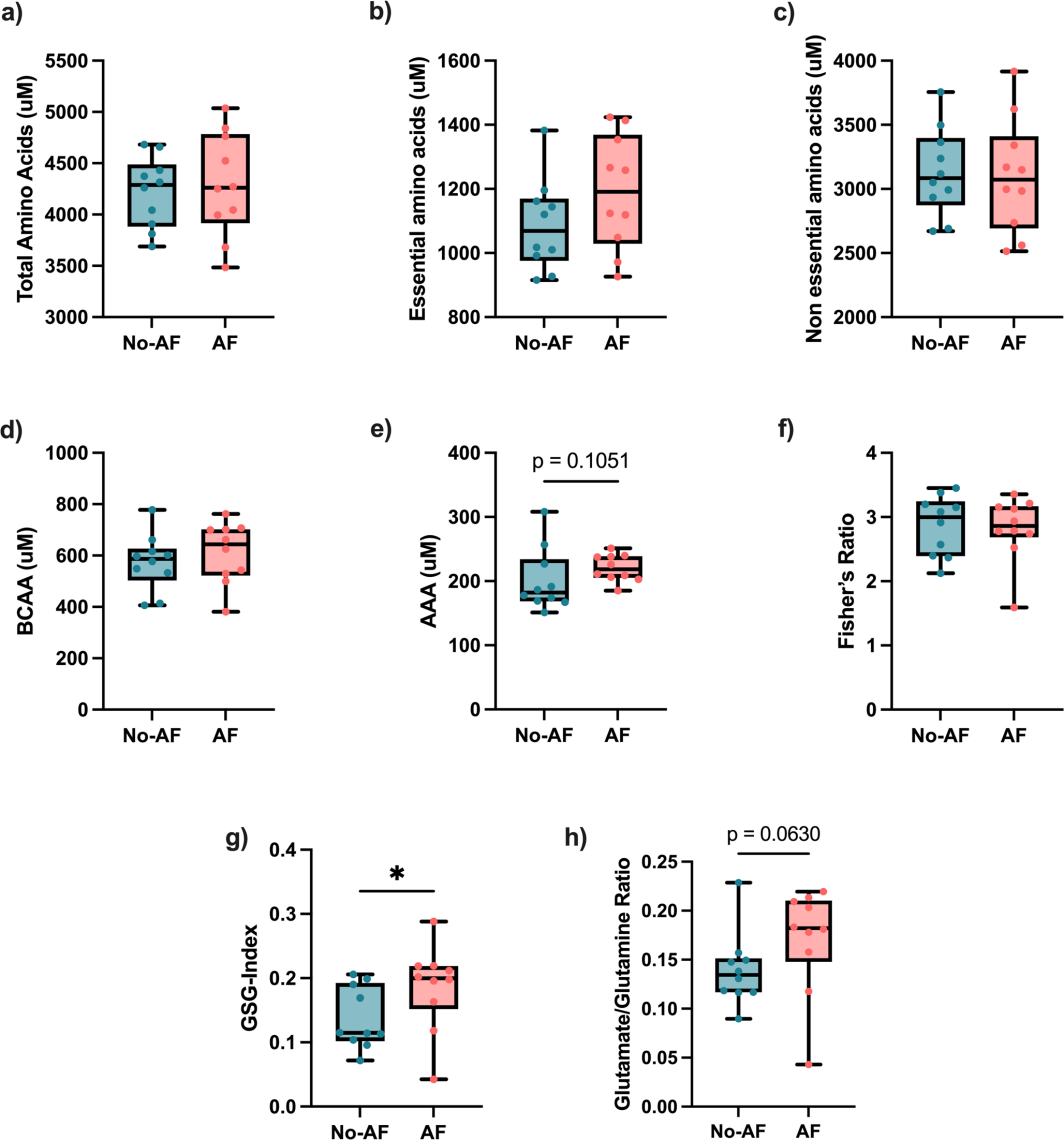
GSG-index and AAA are increased in advanced fibrosis, but not BCAA. Plasmatic concentration of **a** total, **b** essential AA and **c** non-essential AA regarding fibrosis group. **d** BCAA, **e** AAA concentration and **f** ratio between BCAA and AAA in plasma in both groups. **g** GSG-Index and **h** glutamate/glutamine ratio, in accordance with fibrosis stage. Median with interquartile range, *p < 0.05, Mann-Whitney test. *AAA* aromatic amino acids, *AF* advanced fibrosis, *BCAA* branched-chain amino acids; Fisher’s ratio: branched-chain amino acids/aromatic amino acids, *GSG* glutamate-glycine-serine, *No-AF* no advanced fibrosis

Total AAs concentration was unchanged in the AF group compared to the No-AF group (*p* = 0.80, FDR = 0.90) (Fig. 2a). The concentration of essential or non-essential AAs (*p* = 0.25, FDR = 0.74; and *p* = 0.85, FDR = 0.91, respectively) (Fig. 2b and c) was also similar between groups. Concentrations of branched-chain AAs (BCAA) were not influenced by the fibrosis stage (*p* = 0.48, FDR = 0.86) (Fig. 2d), but aromatic AAs (AAA) tended to be higher in the AF group (*p* = 0.1, FDR = 0.65) (Fig. 2e). However, this did not lead to any change in the ratio BCAA/AAA, Fisher’s ratio, reflecting hepatic functional reserve (Sato et al., 2021) (*p* = 0.63, FDR = 0.88) **(**Fig. 2f**)**.

GSG-index, estimated as glutamate/(glycine + serine), was higher in the AF group (*p* < 0.05, FDR = 0.57) **(**Fig. 2g**)**. The glutamate/glutamine ratio, a marker of glutaminolysis (Du et al., 2020), tended to be higher in the AF group (*p* = 0.06, FDR = 0.62) (Fig. 2h).

### TCA cycle intermediates and ketone bodies

**Fig. 3:**
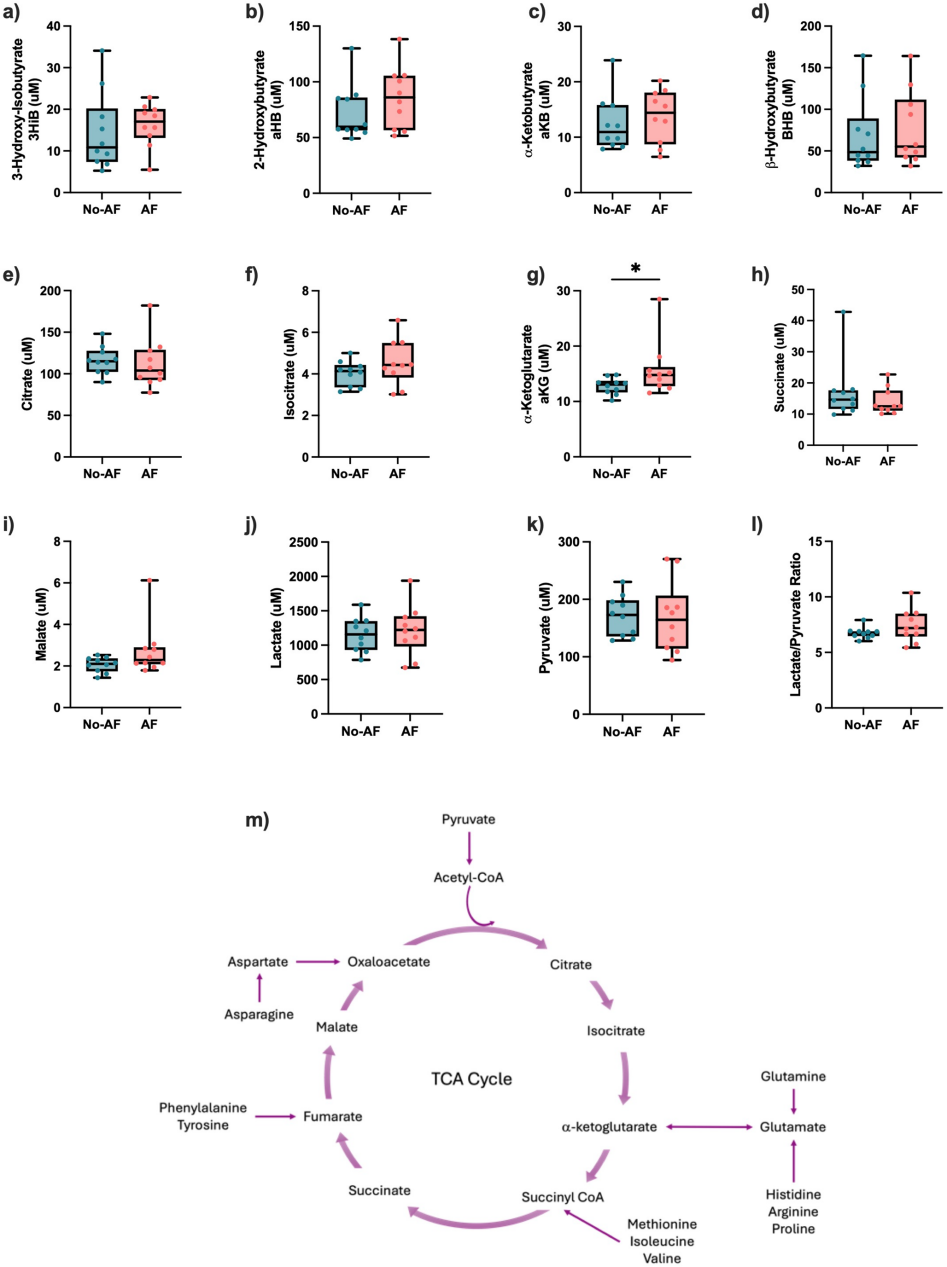
α-ketoglutarate concentration is increased in AF group. Plasma concentration of the following ketones bodies, **a** 3HiB, **b** aHB, **c** α-ketobutyrate and **d** β-hydroxybutyrate. Concentration of the TCA cycle intermediates **e** citrate, **f** isocitrate, **g** α-ketoglutarate, **h** succinate, and **i** malate. **j** lactate, **k** pyruvate and **l** lactate/pyruvate ratio comparing No-AF and AF groups. **m** Anaplerosis and cataplerosis pathway of AA in the TCA cycle, adapted from Owen et al. (Owen et al., 2002). Median with interquartile range, *p < 0.05, **p < 0.01, Mann-Whitney test. *3HiB* 3-hydroxy-isobutyrate, *aHB* 2-hydroxybutyrate, *AF* advanced fibrosis, *aKB* α-ketobutyrate, *aKG* α-ketoglutarate, *BHB* β-hydroxybutyrate, *No-AF* no advanced fibrosis, *TCA* tricarboxylic acid

The presence of AF was not associated with a modification in the plasma concentration of ketone bodies. Concentrations of 3-hydroxy-isobutyrate, 2- hydroxybutyrate, α-ketobutyrate and β-hydroxybutyrate were similar between groups (*p* ≥ 0.25, FDR ≥ 0.74) (Fig. 3a, b, c and d).

Plasma TCA cycle intermediates were also quantified. Citrate and isocitrate were not changed in AF compared to No-AF (*p* ≥ 0.28, FDR ≥ 0.81) (Fig. 3e and f), whereas α-ketoglutarate, was significantly higher in the AF group (*p* < 0.05, FDR = 0.57) (Fig. 3g). Succinate and malate were also similar between groups (*p* ≥ 0.12, FDR ≥ 0.65)** (**Fig. 3h and i). Median [25–75] concentration of each metabolite is presented in Supplemental Table S3 regarding the stage of fibrosis.

Neither lactate nor pyruvate concentrations, nor the lactate/pyruvate ratio, were associated with the presence of advanced fibrosis (*p* ≥ 0.25, FDR ≥ 0.74) (Fig. 3j, k and l).

### Plasma acylcarnitines

**Fig. 4:**
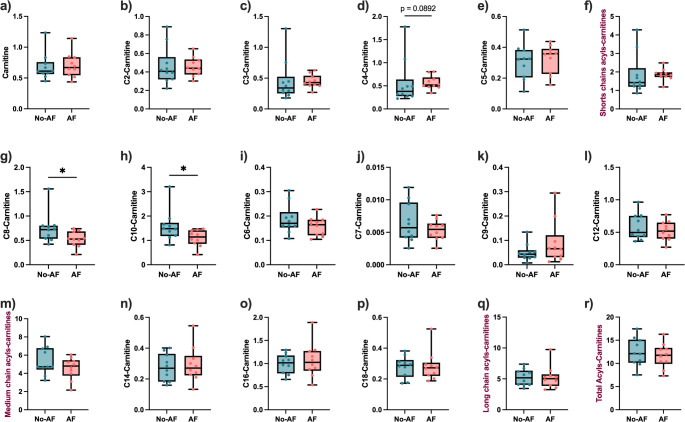
Medium-chain ACs C8 and C10 are decreased in AF. The relative abundance of short-chain ACs **a** Carnitine, **b** C2, **c** C3, **d** C4, **e** C5, and **f** sum of the short-chain ACs in No-AF and AF groups. The medium-chain ACs relative abundance for **g** C8, **h** C10, **i** C6, **j** C7, **k** C9, **l** C12 carnitines and **m** sum of all medium-chain ACs in both groups. **n** C14, **o** C16, **p** C18, and **q** sum of long-chain ACs in both groups. **r** Total ACs according to fibrosis stage. Median with interquartile range, *p < 0.05, Mann-Whitney test. *ACs* acylcarnitines, *AF* advanced fibrosis, *No-AF* no advanced fibrosis

Free carnitine, as well as short-chains C2 and C3 carnitines, were unchanged in the AF group compared to No-AF (*p* ≥ 0.25, FDR ≥ 0.74) (Fig. 4a, b and c). The C4-carnitine tended to be higher without reaching statistical significance in the AF group (*p* = 0.09, FDR = 0.62) (Fig. 4d), whereas the C5-carnitine was similar (*p* = 0.44, FDR = 0.85) (Fig. 4e). The sum of all short-chain ACs was also unchanged between groups (*p* = 0.14, FDR = 0.68) (Fig. 4f).

Medium-chain ACs C8 and C10 carnitines relative abundances were lower in the AF groups (*p* < 0.05, FDR = 0.57 for both) (Fig. 4g and h), whereas C6, C7, C9 and C12-carnitines relative abundance were similar between groups (Fig. 4i, j, k and l). Despite the lower level of two medium-chain ACs, no difference was observed in the total medium-chain ACs between AF and No-AF (*p* = 0.32, FDR = 0.81) (Fig. 4m).

None of the long-chain ACs relative abundances were different between the two groups. That included C14, C16, and C18 carnitines (*p* ≥ 0.68, FDR = 0.90) (Fig. 4n, o and p), and the sum of long-chain Acs (*p* = 0.74, FDR = 0.90) (Fig. 4q).

All ACs relative abundances are presented in Supplemental Table S4.

## Discussion

In this proof-of-concept cross-sectional clinical study, we aimed to describe changes in plasma AAs, TCA cycle intermediates, and ACs profiles in advanced liver fibrosis (F3-F4) without T2D. This aimed to assess energy metabolic pathways and markers of mitochondrial function in advanced stages of MASLD. We observed that participants with advanced fibrosis had higher plasma tyrosine and α-ketoglutarate concentrations and tended to have higher hydroxyproline, asparagine, and methionine concentrations. This suggests increased plasma substrates with anaplerotic potential in advanced liver fibrosis. We found lower level of medium-chain ACs in advanced fibrosis, which could indicate a decrease in β-oxidation and reflect mitochondrial or peroxisomal dysfunction. We also found a higher GSG index, which combines AAs involved in glutathione metabolism; however, we did not measure glutathione itself, and mechanistic interpretation regarding antioxidant synthesis is therefore limited. These results support the general hypothesis that advanced fibrosis in MASLD is associated with increased anaplerotic pathways, increased oxidation and mitochondrial dysfunction, in the absence of T2D, a major confounder, but the small sample size and multiple comparison limit our ability to draw clear conclusions.

### Modification in the amino acid profile in advanced fibrosis

Literature shows consistently that BCAA concentrations are higher in early stages of MASLD (Van Den Berg et al., 2019), with a decrease in the cirrhosis stage (Adeva et al., 2012; Michitaka et al., 2010). However, our study contradicts this hypothesis, as we did not find any differences with AF on plasma BCAA. This could be explained by the absence of T2D and the similar insulin resistance between our groups, with which BCAA are closely associated (Van Den Berg et al., 2019), and may reflect the absence of association with hepatic fibrosis per se. Higher plasma BCAA concentrations have been described as a potential predictor of insulin resistance and onset of T2D (Flores-Guerrero et al., 2018; Wang et al., 2011; Würtz et al., 2013). According to our results, advanced fibrosis alone doesn’t seem to be associated with impaired muscular catabolism of BCAA, which may instead be related to IR and T2D.

Among AAs, we hypothesized that AF would be associated with higher tyrosine concentration, as already described in MASLD and other cirrhosis aetiologies (Masoodi et al., 2021; Miwa Kawanaka et al., 2015; Morgan et al., 1982), which was replicated in our study. Indeed, tyrosine is an AAA, catabolized by the liver and has anaplerotic potential. Importantly, increased plasma tyrosine availability could lead to increased glucogenic and ketogenic pathways in the liver, which could be at the cornerstone of MASLD hepatic oxidative stress and liver fibrosis, as well as increased glucose dysmetabolism. In adolescents with obesity and hepatic steatosis, tyrosine metabolism was the most dysregulated pathway in an untargeted metabolomic study, leading to increased tyrosine abundance in comparison to adolescents without hepatic steatosis (Jin et al., 2016). This means that impaired tyrosine metabolism may appear early in the disease. Liver tyrosine, phenylalanine and BCAA concentrations were also shown to be elevated in the liver of patients with MASH in comparison to simple hepatic steatosis. Our results suggest a concentration of tyrosine even higher in adults with advanced stages of liver fibrosis, in comparison to early stages, independent of impaired glucose metabolism (Lake et al., 2015). As the origin of plasma tyrosine is uncertain, the higher plasma tyrosine concentration could come from higher endogenous muscle protein breakdown (Tessari et al., 2010), as described in MASLD (Isakov, 2025), or from the liver by increased phenylalanine hydroxylation (Tessari et al., 2010). Even if hydroxylation contributes to 10–20% of tyrosine flux, it has been documented to be increased in compensated cirrhosis, such as in our sample, and decreased in end-stage liver disease (Tessari et al., 2010). Tyrosine have an anaplerotic potential through fumarate, but fumarate was unmeasurable in our study, which limits our ability to determine the impact of its increase in anaplerosis.

### α-ketoglutarate is the only TCA cycle intermediate changed in advanced fibrosis

Increased plasma TCA intermediates could inform on mitochondrial anaplerosis/cataplerosis pathways and could reflect liver pathways, being a major contributor to cataplerotic metabolic fluxes through gluconeogenesis (Owen et al., 2002) and fatty acid de novo synthesis. We hypothesized that plasma TCA cycle intermediates would be elevated in the AF group, reflecting this increased anaplerotic/cataplerotic activity. However, only α-ketoglutarate was increased in AF. A higher plasma concentration of isocitrate and citrate in MASLD compared to healthy controls was already described (Sandlers et al., 2020). The absence of MASH and AF participants in Sandlers et al. study could explain the differences observed in the metabolic profile compared to our study. Indeed, in steatosis and MASH, compared to patients without MASLD, a higher plasma level of α-ketoglutarate was observed (Aragonès et al., 2016). α-ketoglutarate can be derived from glutaminolysis, as reflected by the higher glutamate/glutamine ratio found in our study (Du et al., 2020). Glutaminolysis, by increasing α-ketoglutarate, may be involved in fibrosis progression, potentially through the reprogramming of hepatic stellate cells into proliferative, migratory and fibrogenic myofibroblasts (Du et al., 2018). Longitudinal studies should be done to confirm this hypothesis.

### Lower level of medium-chain acylcarnitine with advanced fibrosis

In the opposite of our hypothesis based on studies in MASH vs. controls (Kalhan et al., 2011), we did not find a higher plasma level of short and medium-chain ACs in the group with AF, that has been interpreted as reflecting incomplete β-oxidation. We found, instead, lower C8 and C10 medium-chain ACs. Interestingly, the plasma profile of ACs seems to be closely linked to their hepatic metabolism, with a small contribution from other organs such as muscles (Schooneman et al., 2015). However, retention of ACs in different organelles could lead to a loss of hepatic reflection at the plasma level. AC profiles found in our participants with advanced fibrosis were not clearly consistent with an incomplete β-oxidation, since we found a lower relative abundance of medium-chain ACs. With our results and the literature, several hypotheses could explain our results. The observed lower level of medium-chain ACs could also reflect mitochondrial or peroxisomal dysfunction. Mitochondrial dysfunction has been suggested to occur at the AF stage; however, our data do not provide direct evidence for this mechanism, and plasma lactate/pyruvate does not allow reliable inference on intracellular redox state. Likewise, medium-chain ACs are end-products of peroxisomal fatty acid oxidation; therefore, their decrease in the advanced stage of fibrosis may also be compatible with altered peroxisomal β-oxidation (Houten et al., 2020), but the absence of changes in long and very-long chain AC contradict this hypothesis.

### The ketone bodies profile is similar between the fibrosis groups

We hypothesized a higher concentration of plasma ketone bodies in the AF group. However, we did not find any differences in ketone bodies between groups. Indeed, in a large cohort, patients with MASLD had an higher plasma level of total ketone bodies, β-hydroxybutyrate, acetoacetate and acetone (Post et al., 2021). However, another study showed a lower β-hydroxybutyrate concentration with the increase of NAFLD activity score (Moore et al., 2024). A third study showed that after a 12-hour fast, ketone bodies concentrations were similar between MASLD patients and controls (Fletcher et al., 2019). Ketogenesis may increase in the simple hepatic steatosis stage and then decrease in the advanced form (Mooli & Ramakrishnan, 2022). However, our study could not replicate these results.

### Limitations

As this is a pilot proof-of-concept study, the small sample size potentially did not have enough statistical power to draw some conclusions on several metabolites. Furthermore the use of certain medications, such as metformin or GLP-1 agonists, remain significant confounders in this study. However, we could identify some potential differences that reinforced our hypotheses on altered metabolic pathways that occur in advanced fibrosis. Furthermore, our sample was very homogeneous regarding ethnicity, and participants were all recruited in a specialized clinic in endocrinology, with most of them presenting with comorbidities requiring specialists care, which may limit the generalization of our results.

## Conclusion

We identified that participants with MASLD and an advanced stage of liver fibrosis but without T2D have higher plasma concentrations of tyrosine and α-ketoglutarate, and a lower relative abundance of some medium-chain ACs. These individuals also tended to have higher hydroxyproline, methionine, histidine, asparagine, and glutamate plasma concentrations than individuals with MASLD and no advanced fibrosis. The higher concentration of substrates with anaplerotic potential, with a potential lower β-oxidation, highlighted by the altered profile of acylcarnitines, may reflect a mitochondrial dysfunction and an adaptation of liver energetic pathways. More studies are necessary to delineate the impact of advanced fibrosis on plasma metabolic profiles.

## Supplementary Information

Below is the link to the electronic supplementary material.

Supplementary Material 1.

## Data Availability

The data supporting the findings of this study are available from the corresponding author upon reasonable request.
