# Supplementary material for: Metabolomic and lipidomic plasma profiles according to metabolic dysfunction-associated steatotic liver diseases (MASLD) stages in the absence of type 2 diabetes (T2D)
Source: Metabolomics. 2026 May 7;22(3):67. doi: 10.1007/s11306-026-02437-1 (PMC13152879; doi:10.1007/s11306-026-02437-1)
Supplement: Supplementary file 1 — Supplementary Material 1. [file 11306_2026_2437_MOESM1_ESM.docx]

Supplemental Table S1. Quality controls data for the metabolomic platform

| Metabolites | Ion (m/z) | RT (min) | Internal Standard | LOQ (nmol) | Intra-day % CV | Recovery (%) |
| --- | --- | --- | --- | --- | --- | --- |
| Alanine | 263 | 14.86 | U-^13^C_3_-alanine | 0.42 | 4.70 | 98.0 |
| Arginine | 448 | 30.85 | U-^13^C_6_-arginine | 0.20 | 3.39 | 101.5 |
| Asparagine | 423 | 28.42 | U-^13^C_4_,U-^15^N_2_-asparagine | 0.50 | 2.96 | 94.2 |
| Aspartate | 423 | 26.32 | U-^13^C_4_,U-^15^N aspartate | 0.13 | 11.72 | 96.7 |
| Cysteine | 410 | 27.14 | U-^13^C_3_,^15^N-cystéine | 37.47 | 12.69 | 105.3 |
| Glutamate | 438 | 27.91 | ^13^C_5_,^15^N-glutamate | 2.50 | 0.51 | 0.4.4 |
| Glutamine | 438 | 29.99 | U-^13^C_5_,U-^15^N glutamine | 0.98 | 9.39 | 83.0 |
| Glycine | 248 | 15.25 | ^13^C_2_-glycine | 1.98 | 0.71 | 101.8 |
| Histidine | 446 | 32.62 | U-^13^C_6_-histidine | 0.90 | 2.86 | 106.8 |
| Hydroxyproline | 292 | 19.99 | U-^13^C_5_,^15^N-proline | 1.64 | 6.62 | 100.2 |
| Isoleucine | 308 | 19.05 | U-^13^C_6_-isoleucine | 0.13 | 1.31 | 94.2 |
| Leucine | 309 | 18.35 | U-^13^C_6_,^15^N-leucine | 0.15 | 2.33 | 102.1 |
| Lysine | 439 | 29.34 | U-^13^C_6_,U-^15^N_2_-lysine | 0.80 | 2.88 | 78.4 |
| Methionine | 325 | 23.51 | U-^13^C_5_-méthionine | 0.02 | 3.52 | 90 |
| Phenylalanine | 341 | 23.56 | d_5_-phénylalanine | 0.26 | 4.89 | 105.7 |
| Proline | 292 | 19.99 | U-^13^C_5_,^15^N-proline | 0.18 | 2.81 | 90 |
| Serine | 394 | 23.82 | U-^13^C_3_,^15^N-serine | 0.80 | 2.80 | 85.5 |
| Threonine | 409 | 24.41 | U-^13^C_4_,^15^N-threonine | 0.40 | 2.49 | 98.6 |
| Tryptophane | 388 | 34.48 | U-^13^C_11_,U-^15^N tryptophane | 0.30 | 3.59 | 98.3 |
| Tyrosine | 475 | 33.32 | U-^13^C_9_-tyrosine | 011 | 4.96 | 86 |
| Valine | 293 | 17.56 | U-^13^C_5_-valine | 0.27 | 1.75 | 100.5 |
| 2-Hydroxybutyrate | 264 | 13.4 | U-^13^C sodium lactate | 7.56 | 11.5 | 102.8 |
| 3-Hydroxy-Isobutyrate | 264 | 13.4 | U-^13^C sodium lactate | 0.55 | NA | 97 |
| α-Ketobutyrate | 294 | 16.1 | ^13^C_4_,d_2_-α-ketobutyrate | 0.012 | 9.0 | 100.4 |
| β-Hydroxybutyrate | 279 | 15.46 | ^13^C_4_-β-hydroxybutyrate | 0.11 | 6.2 | 88.8 |
| Citrate | 462 | 32.11 | d_4_-citrate | 0.3 | 9.1 | 113.8 |
| Isocitrate | 462 | 32.11 | d_4_-citrate | 0.08 | 5.2 | 98.1 |
| α-ketoglutarate | 450 | 27.54 | ^13^C_4_-α-ketoglutarate | 0.02 | 15.1 | 100.3 |
| Lactate | 264 | 13.4 | U-^13^C sodium lactate | 6.23 | 13.2 | 97.9 |
| Malate | 422 | 25.1 | d_3_-malate | 1.03 | 10.8 | 97.2 |
| Pyruvate | 277 | 15.27 | U-^13^C sodium pyruvate | 0.2 | 13.6 | 99.6 |
| Succinate | 293 | 18.90 | d_4_-succinate | 4.18 | 4.1 | 100 |

Supplemental Table S2. Plasma amino acid concentration according to liver fibrosis stage

| Amino acids (μM) | Median [25-75] | | p value | FDR |
| --- | --- | --- | --- | --- |
|  | No-Advanced fibrosis (No-AF) | Advanced Fibrosis (AF) |  |  |
| Alanine | 415.09 [401.29-425.19] | 426.93 [409.92-481.87] | 0.53 | 0.88 |
| Arginine | 332.76 [296.16-352.36] | 315.03 [295.26-341.29] | 0.74 | 0.90 |
| Asparagine | 42.67 [40.67-44.65] | 49.88 [44.12-57.73] | 0.09 | 0.62 |
| Aspartate | 2.28 [2.07-3.16] | 2.65 [2.27-3.04] | 0.58 | 0.88 |
| Cysteine | 301.3 [244.59-321.24] | 282.13 [262.77-286.04] | 0.97 | >0.99 |
| Glutamate | 106.31 [88.79-128.92] | 132.01 [113.36-148.77] | 0.11 | 0.65 |
| Glutamine | 808.58 [746.17-845.84] | 800.41 [693.98-867.78] | 0.97 | >0.99 |
| Glycine | 655.61 [598.04-731.31] | 657.58 [496.72-668.4] | 0.53 | 0.88 |
| Histidine | 81.06 [80.19-87.08] | 89.09 [86.7-96.66] | 0.11 | 0.65 |
| Hydroxyproline | 3.95 [3-4.47] | 5.21 [4.5-6.57] | 0.08 | 0.32 |
| Isoleucine | 63.5 [59.78-76.52] | 77.04 [65.13-86.48] | 0.22 | 0.74 |
| Leucine | 131.49 [119.95-139.79] | 151.74 [124.22-168.09] | 0.44 | 0.85 |
| Lysine | 160.87 [148.8-176.01] | 184.74 [155.41-214.25] | 0.19 | 0.74 |
| Methionine | 23.77 [19.92-25.2] | 27.35 [25.45-29.71] | 0.09 | 0.62 |
| Phenylalanine | 61.92 [56.37-66.83] | 66.42 [62.22-72.36] | 0.25 | 0.7 |
| Proline | 230.96 [208.16-254.69] | 223.45 [175.75-234.6] | 0.69 | 0.90 |
| Serine | 114.2 [101.47-126.42] | 139.54 [103.86-149.57] | 0.25 | 0.74 |
| Threonine | 122.73 [107.56-143.66] | 152.29 [120.87-170.74] | 0.17 | 0.72 |
| Tryptophane | 56.99 [51.81-71.46] | 63.06 [56.07-64.95] | 0.68 | 0.90 |
| Tyrosine | 63.78 [61.97-81.56] | 93.62 [82.57-100.1] | **0.04** | 0.57 |
| Valine | 375.28 [355.56-393.14] | 414.71 [339.51-441.77] | 0.74 | 0.90 |

Supplemental Table S3. Plasma TCA Cycle intermediaries and ketone bodies concentration according to liver fibrosis stage

| Metabolites (μM) | Median [25-75] | | p value | FDR |
| --- | --- | --- | --- | --- |
|  | No-Advanced fibrosis (No-AF) | Advanced Fibrosis (AF) |  |  |
| 3-Hydroxy-Isobutyrate | 10.86 [7.95-17.5] | 17.06 [14.17-19.71] | 0.25 | 0.74 |
| 2-Hydroxybutyrate | 59.64 [57.14-85.02] | 86.05 [61.05-104.28] | 0.35 | 0.81 |
| α-Ketobutyrate | 10.94 [8.93-14.83] | 14.41 [10.01-17.58] | 0.48 | 0.86 |
| β-Hydroxybutyrate | 48.43 [39.89-74.5] | 55.27 [44.22-102.85] | 0.63 | 0.88 |
| Citrate | 115.04 [105.32-124.01] | 103.98 [93.84-125.19] | 0.44 | 0.85 |
| Isocitrate | 4.14 [3.52-4.33] | 4.43 [4.11-5.23] | 0.28 | 0.81 |
| α-Ketoglutarate | 13.06 [11.87-13.41] | 14.77 [13.17-15.49] | **0.04** | 0.57 |
| Succinate | 14.64 [12.15-17.38] | 12.54 [11.43-15.91] | 0.48 | 0.86 |
| Malate | 2.1 [1.85-2.33] | 2.29 [2.13-2.84] | 0.12 | 0.65 |
| Lactate | 1157.76 [957.41-1329.02] | 1222.01 [1077.5-1377.46] | 0.74 | 0.90 |
| Pyruvate | 172.79 [138.42-194.13] | 164.37 [119.57-186.24] | 0.63 | 0.88 |

Supplemental Table S4. Plasma Acylcarnitines ratios according to liver fibrosis stage

| Acyl-carnitines (relative abundance) | Median [25-75] | | p value | FDR |
| --- | --- | --- | --- | --- |
|  | No-Advanced fibrosis (No-AF) | Advanced Fibrosis (AF) |  |  |
| Carnitine | 0.61 [0.58-0.72] | 0.67 [0.58-0.81] | 0.63 | 0.88 |
| C2 Carnitine | 0.41 [0.34-0.48] | 0.44 [0.39-0.52] | 0.58 | 0.88 |
| C3 Carnitine | 0.34 [0.27-0.44] | 0.43 [0.39-0.51] | 0.25 | 0.74 |
| C4 Carnitine | 0.38 [0.29-0.49] | 0.52 [0.48-0.63] | 0.09 | 0.62 |
| C4-OH Carnitine | 0.03 [0.02-0.03] | 0.03 [0.02-0.05] | 0.39 | 0.84 |
| C5:1 Carnitine | 0.02 [0.01-0.03] | 0.02 [0.02-0.02] | 0.39 | 0.84 |
| C5 Carnitine | 0.32 [0.23-0.37] | 0.36 [0.26-0.38] | 0.44 | 0.85 |
| C6:1 Carnitine | 0.01 [0.01-0.02] | 0.02 [0.01-0.02] | 0.48 | 0.86 |
| C6 Carnitine | 0.17 [0.16-0.2] | 0.16 [0.12-0.18] | 0.31 | 0.81 |
| C6-OH Carnitine | 0.02 [0.01-0.02] | 0.02 [0.01-0.03] | 0.58 | 0.88 |
| C7 Carnitine | 0.01 [0-0.01] | 0.01 [0-0.01] | 0.53 | 0.88 |
| C8:1 Carnitine | 0.98 [0.61-1.22] | 1.04 [0.76-1.24] | 0.68 | 0.90 |
| C8 Carnitine | 0.72 [0.56-0.8] | 0.53 [0.44-0.65] | **0.04** | 0.57 |
| C8-OH Carnitine | 0.1 [0.09-0.12] | 0.12 [0.1-0.14] | 0.44 | 0.85 |
| C9 Carnitine | 0.04 [0.03-0.05] | 0.07 [0.03-0.09] | 0.35 | 0.81 |
| C10:2-Carnitine | 0.07 [0.06-0.1] | 0.07 [0.06-0.1] | >0.99 | >0.99 |
| C10:1 Carnitine | 0.65 [0.46-0.77] | 0.51 [0.45-0.59] | 0.31 | 0.81 |
| C10 Carnitine | 1.48 [1.24-1.64] | 1.14 [0.92-1.37] | **0.04** | 0.57 |
| C10-OH Carnitine | 0.08 [0.07-0.1] | 0.08 [0.07-0.11] | >0.99 | >0.99 |
| C12:2 Carnitine | 0.05 [0.04-0.05] | 0.04 [0.03-0.06] | 0.97 | >0.99 |
| C12:1 Carnitine | 0.19 [0.14-0.22] | 0.2 [0.15-0.23] | 0.80 | 0.90 |
| C12 Carnitine | 0.5 [0.44-0.73] | 0.52 [0.42-0.63] | 0.74 | 0.90 |
| C14:2 Carnitine | 0.22 [0.17-0.27] | 0.2 [0.16-0.22] | 0.53 | 0.88 |
| C14:1 Carnitine | 0.82 [0.6-1.05] | 0.83 [0.6-0.91] | 0.80 | 0.90 |
| C14 Carnitine | 0.27 [0.19-0.34] | 0.27 [0.23-0.32] | 0.80 | 0.90 |
| C14:1-OH Carnitine | 0.03 [0.03-0.04] | 0.04 [0.03-0.04] | 0.63 | 0.88 |
| C14-OH Carnitine | 0.04 [0.03-0.05] | 0.05 [0.04-0.06] | 0.35 | 0.81 |
| C16:2 Carnitine | 0.05 [0.04-0.07] | 0.05 [0.04-0.05] | 0.85 | 0.91 |
| C16:1 Carnitine | 0.31 [0.27-0.43] | 0.34 [0.26-0.39] | 0.80 | 0.90 |
| C16 Carnitine | 1.02 [0.84-1.14] | 1.03 [0.88-1.24] | 0.85 | 0.91 |
| C18:3 Carnitine | 0.03 [0.02-0.04] | 0.04 [0.03-0.04] | 0.85 | 0.91 |
| C18:2 Carnitine | 0.41 [0.36-0.51] | 0.35 [0.34-0.56] | 0.48 | 0.86 |
| C18:1 Carnitine | 1.56 [1.26-1.87] | 1.34 [1.21-1.85] | 0.63 | 0.88 |
| C18 Carnitine | 0.29 [0.24-0.31] | 0.27 [0.23-0.29] | 0.68 | 0.90 |
| C20:4 Carnitine | 0.02 [0.02-0.02] | 0.02 [0.01-0.03] | 0.91 | 0.97 |
| C20:3 Carnitine | 0.02 [0.02-0.03] | 0.02 [0.02-0.02] | 0.35 | 0.81 |
| C20:2 Carnitine | 0.02 [0.02-0.03] | 0.02 [0.02-0.02] | 0.74 | 0.90 |
